# Multiscale Enhanced Sampling Using Machine Learning

**DOI:** 10.3390/life11101076

**Published:** 2021-10-12

**Authors:** Kei Moritsugu

**Affiliations:** Graduate School of Medical Life Science, Yokohama City University, Yokohama 230-0045, Japan; moritugu@yokohama-cu.ac.jp; Tel.: +81-45-508-7233

**Keywords:** enhanced sampling, multiscale enhanced sampling (MSES), machine learning, variational autoencoder (VAE), ribose-binding protein

## Abstract

Multiscale enhanced sampling (MSES) allows for an enhanced sampling of all-atom protein structures by coupling with the accelerated dynamics of the associated coarse-grained (CG) model. In this paper, we propose an MSES extension to replace the CG model with the dynamics on the reduced subspace generated by a machine learning approach, the variational autoencoder (VAE). The molecular dynamic (MD) trajectories of the ribose-binding protein (RBP) in both the closed and open forms were used as the input by extracting the inter-residue distances as the structural features in order to train the VAE model, allowing the encoded latent layer to characterize the difference in the structural dynamics of the closed and open forms. The interpolated data characterizing the RBP structural change in between the closed and open forms were thus efficiently generated in the low-dimensional latent space of the VAE, which was then decoded into the time-series data of the inter-residue distances and was useful for driving the structural sampling at an atomistic resolution via the MSES scheme. The free energy surfaces on the latent space demonstrated the refinement of the generated data that had a single basin into the simulated data containing two closed and open basins, thus illustrating the usefulness of the MD simulation together with the molecular mechanics force field in recovering the correct structural ensemble.

## 1. Introduction

The construction of the free energy surface (FES) from a well-converged structural ensemble is the ultimate goal of computational biophysics in understanding biomolecular functions. To this aim, various kinds of enhanced sampling methods have been developed [1,2,3,4,5,6,7,8] since it would be difficult for a straightforward application of a brute-force molecular dynamic (MD) simulation to cover the whole configurational space. Despite recent advances in supercomputing and GPU hardware, there remains a limitation in the application of the methods to large biomolecules, especially when a number of comparative systems need to be simulated such as the binding of different chemical compounds.

Previous works have proposed the multiscale enhanced sampling (MSES) in which the sampling of the target protein model at an atomistic resolution is enhanced by coupling with the accelerated dynamics of the associated coarse-grained (CG) model [9,10,11,12,13,14,15,16,17,18]. The multiscale simulation system comprises an all-atom model of proteins with its surrounding solvents together with the molecular mechanics (MM) force field (**r**_MM_; *N* degrees of freedom) and a CG model of the proteins (**r**_CG_; *M* degrees of freedom). The potential energy is given by the following equation:

(1) $$V=V_{\mathrm{MM}}\left(r_{\mathrm{MM}}\right)+V_{\mathrm{CG}}\left(r_{\mathrm{CG}}\right)+k_{\mathrm{MMCG}}{\left[\chi_{\mathrm{MM}}\left(r_{\mathrm{MM}}\right)-\chi_{\mathrm{CG}}\left(r_{\mathrm{CG}}\right)\right]}^{2}\left(\equiv V_{\mathrm{MMCG}}\right),$$

where *V*_MM_ and *V*_CG_ are the MM and CG potential energy functions, respectively. *V*_MMCG_ is the coupling term between MM and CG with a coupling constant, *k*_MMCG_, which is useful for accelerating the MM dynamics via coupling with CG. **χ**_CG_(**r**_CG_) is defined by the *K* collective variables of the CG coordinates, and **χ**_MM_(**r**_MM_) is a *K*-dimensional vector that is a projection of **r**_MM_ onto the associated *K*-dimensional space. The intrinsic free energy surface solely from *V*_MM_ can be obtained by eliminating bias from the coupling *V*_MMCG_ via Hamiltonian replica exchange in which many replicated systems are assigned various values of *k*_MMCG_ that range from a large value to zero [19]. The condition *K* < *M* << *N* ensures a high exchange probability irrespective of the size of MM, *N*, which achieves excellent scalability that is applicable to large protein systems in solution [9]. Moreover, the approximation of an adiabatic separation [20] allows for further efficient sampling by using a single copy of CG that does not experience counterforce from MM, while replicated MM systems are simulated as the restraint MDs between **χ**_MM_(**r**_MM_) and **χ**_CG_(**r**_CG_) with various *k*_MMCG_ values [13].

In MSES, *V*_CG_ can be arbitrarily chosen based on prior knowledge or experimental data, depending on which subspace is selected for enhanced sampling. However, constructing a CG model that requires the proper selection of a set of parameters is a difficult task, especially for those who are not familiar with computation. In this article, an extension that utilizes a machine learning approach instead of adopting the CG model is proposed—the variational autoencoder (VAE) [21]. VAE is a (deep) neural network that learns encodings for the input data by trying to reconstruct high-dimensional data through low-dimensional encoded data in latent space. This is also a complex generative model of data through the latent space as a representation of compressed data, which is then useful for not only image generation and pattern recognition, but also for the systematic discovery of data-driven collective variables (CVs) from biomolecular simulation data [22,23,24,25,26]. The proper selections of nonlinear CVs is expected in an automatic manner, which is expected to be more efficient than the methods that use linear CVs such as principal component (PC) analysis.

**Figure 1:**
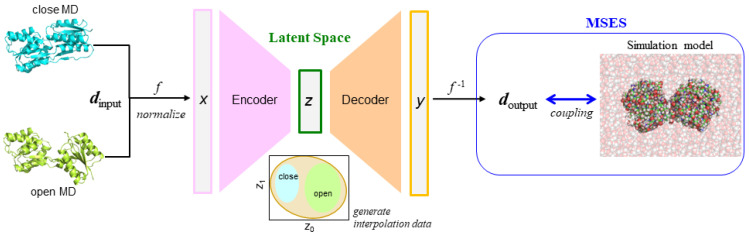
Scheme of VAE-driven MSES. Two trajectories of the closed and open MDs were used as input ***x*** for encoding, after normalizing the associated inter-residue distances ***d***_input_ via *f*. In the latent space ***z***, the interpolated data between the closed and open forms were generated and then decoded to ***y***, allowing for *f*^−1^(***y***) = ***d***_output_ to be calculated. ***d***_output_ was thus used to drive the sampling of the simulation model such as the all-atom protein structure, including explicit solvent.

The present application of VAE to MSES, termed as “VAE-driven MSES”, is summarized as follows (Figure 1): suppose that there are two different structures, such as closed and open conformations, and that the enhanced sampling is aimed at interpolating the two structures. The MD simulations starting at the two structures are performed and the trajectories of both the closed MD and open MD are used as inputs of the network. Inter-residue distances ***d***_input_ are used as the structural features, motivated by the recent successes of high-quality protein structure prediction such as AlphaFold [27]. The input ***x*** needs to be normalized via *f* as the values from 0 to 1. Since the latent layer encodes information regarding the difference in the structural dynamics of the closed and open forms, the interpolated data can be efficiently generated in the latent space ***z***. The generated data is then decoded to output ***y***, allowing for *f*^−1^(***y***) = ***d***_output_ to be calculated. ***d***_output_ is then used as the distance restraints on the MM simulations, thus driving the MM conformational sampling via the scheme of MSES.

The proposed method was applied to the structural change of the ribose-binding protein (RBP). RBP is a periplasmic protein that binds ribose and undergoes an open-close motion of the two ligand-binding domains between the ligand-bound/closed form and the ligand-unbound/open form. The validity of VAE-driven MSES was demonstrated by comparing the derived RBP structural ensemble with that from the original MSES adopting the CG model. The dynamics in the latent space of VAE was also examined to show how the generated data in the reduced space could work for the enhanced sampling. The extrapolation or prediction of the open structure using only the MD trajectories in the closed form with and without the ligand (i.e., when the open structure was blind) was also attempted, which illustrates the usefulness of the MSES simulation together with the molecular mechanics force field in refining the generated data via VAE so as to recover the correct structural ensemble.

## 2. Materials and Methods

### 2.1. Model Construction and MD Simulation

MD simulations were carried out for RBP with and without a ribose molecule as the ligand. The present study aimed at an enhanced sampling in the ligand-unbound form, i.e., for large-scale structural change seen in two crystal structures—the ligand-bound/closed form and the ligand-unbound/open form, since the dynamics in the ligand-bound form is considered to be within the fluctuation around the closed structure. To prepare for the subsequent enhanced sampling, three MD simulations were performed, in as short as 100 ns each, for (1) the ligand-bound/closed form (termed herein as “holo”), (2) the ligand-unbound/closed form (termed herein simply as “close”), and (3) the ligand-unbound/open form (termed herein as “open”). The holo and open simulation models were taken from the crystal structures of the Protein data Bank (PDB) entries 2dri [28] and 1ba2 [29], respectively. The R67D point mutation in the holo form was converted into a wild type. The model of the closed simulation was constructed by removing the ligand from the holo model. For the three models, rectangular simulation boxes were constructed, with a margin of 12 Å from the boundary of the simulation box, fully solvated by 20,000–25,000 TIP3P water molecules [30] and potassium and sodium ions at a concentration of ~150 mM, resulting in 70,000–80,000 atoms in total. AMBER ff14SB [31] was used for the potential energy of the protein, and GLYCAM06 [32] for β-D-ribopyranose. The MD simulations were performed by AMBER 16 [33] under constant temperature and pressure (NPT) conditions at *P* = 1 atm and *T* = 300 K, using a Berendsen’s barostat and Langevin dynamics to control the temperature settings and 1.0 ps^−1^ as the collision frequency. The particle mesh Ewald method [34] was employed for the electrostatic interactions. The time step was 2 fs, using constraining bonds that involve hydrogen atoms via the SHAKE algorithm [35]. The three MD simulation trajectories were used for analyses that were taken every 10 ps.

### 2.2. Motion Tree

Protein dynamics can be simplified by a set of inter-domain motions once the number of domains is determined, which are then taken as rigid structural units. The regions of such domains are defined by the hierarchical clustering of inter-residue distance fluctuation to construct a tree diagram named “Motion Tree” [36]. The Motion Tree illustrates a pair of domains and the magnitude of the associated domain motion at each node in a hierarchical manner. In previous studies, the Motion Trees were calculated and utilized to analyze complicated MD simulation trajectories [37,38,39] as well as crystal structure ensembles [14].

The distance fluctuation as a metric of the hierarchical clustering is calculated as:

(2) $$D_{mn}=\langle \Delta d_{mn}^{2}\rangle {}^{1/2},$$

where Δ*d*_mn_ is the distance between the Cα atoms of residues *m* and *n*, and <…> is the average over the structural ensemble. Here, both the closed and open MD simulation trajectories were used as the structural ensemble for constructing the Motion Tree. The inter-residue distances between the two defined domains (domain 1 and domain 2) were then used as the features or the input variables of the subsequent VAE (see below), instead of using all the inter-residue distances in an inefficient manner.

### 2.3. Variational Autoendcoder

In this study, the VAE architecture used consisted of seven fully connected layers containing *N*_inp_, 1000, 1000, *L*, 1000, 1000, and *N*_inp_ nodes (three layers in both encoder and decoder parts, and a coding layer comprising *L* nodes). Test calculations using different numbers of layers and nodes were found to yield similar results. As the input data, the inter-residue distances (between Cα atoms; ***d***_input_) for domain 1 (114 residues) and 2 (131 residues), excluding 1–2 (virtual bond) and 1–3 (virtual angle) interactions, were chosen (see Results below), i.e., the number of elements for ***d***_input_ was *N*_inp_ = 14,448. The normalization for the input data was calculated as follows:

(3) $$x=f\left(d_{\mathrm{input}}\right)=0.4\left(1/d_{\mathrm{input}}-0.14\right),$$

where ***d*** is in the unit of [nm]. Here, the inverse of ***d*** was taken, since closer inter-residue distances will have more information on the structure description such as the atom contacts. Other choices for the input data and *f* that can make the neural network more efficient and interpretable are also possible, and will be studied in future work. The model was trained by optimizing the sum of reconstruction loss and the Kullback–Leibler divergence for 300 epochs using the Adam optimizer, which was implemented using PyTorch v1.9.1 (https://pytorch.org/, accessed on 12 October 2021). The training data of 20,000 structures were taken from two 100 ns trajectories of the closed and open MDs. The coding of the python script was described by [21].

### 2.4. Multiscale Enhanced Sampling

The VAE-driven MSES simulation was performed for RBP in the ligand-unbound form. The *V*_MM_ was the same as in the MD simulations. The coupling potential *V*_MMCG_ in Equation (1) was replaced by the harmonic distance constraints of ***d***_output_(*t*) that were imposed on the *N*_inp_ = 14,448 Cα atom pairs between the two dynamic domains. The Hamiltonian replica exchange was carried out every 40 ps using 10 replicas with *k*_MMCG_ = 0, 0.000008, 0.000014, 0.000025, 0.000043, 0.000072, 0.000119, 0.000194, 0.000312, and 0.00049 kcal/mol/Å^2^. The number of replica exchanges was 5000, corresponding to a 200 ns simulation. The trajectories of the unbiased (*k*_MMCG_ = 0) replica were taken as the target structural ensemble that were used for analyses.

For comparison, the original MSES using the CG model was also carried out. The 271 Cα atoms in RBP were chosen as the CG coordinates. *V*_CG_ was set as the double-well model that embedded the two closed and open crystal structures so as to drive the structural changes between the two structures [40]. The CG simulation was performed using Langevin dynamics, with a friction constant of 1 ps^−1^ under constant temperature conditions (NVT) of *T* = 1000 K in order to satisfy the condition of adiabatic separation, with a heavy mass of 10,000 amu [13]. The other parameters on the MSES simulation were the same as described above for the VAE-driven MSES. The MSES simulations were performed with my own script.

## 3. Results and Discussion

### 3.1. MD Trajectory Analysis for Processing VAE Input Data

The short-time (100-ns) MD simulations of RBP in both the closed and open forms were performed in order to generate the training data for VAE to optimize the latent space, which characterized the difference between the two structural dynamics. Since the success of the neural network will rely strongly on the processing of the input data, the MD simulation trajectories were firstly analyzed in detail to find out the structural features that were useful in describing the RBP inter-domain motion.

**Figure 2:**
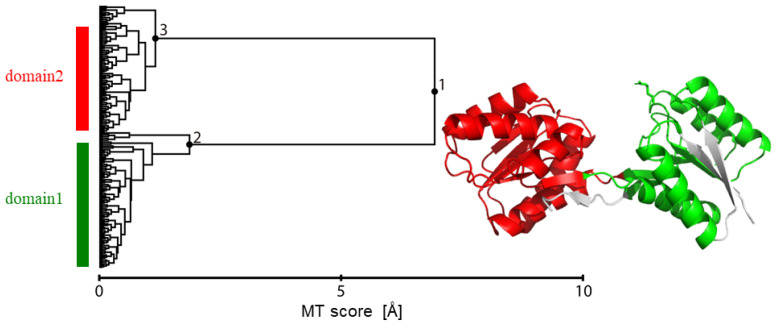
Motion Tree constructed from two trajectories of the closed MD and open MD. This defines two dynamic domains (shown on the right panel in green: residues 10–30, 39–102, 234–262, and in red: residues 103–233) that move like rigid bodies, after removing flexible regions defined at Nodes 2 and 3.

To do this, the Motion Tree was calculated from the closed and open MD trajectories (see Methods). The Tree illustrated clear definitions of the two dynamic domains at Node 1 that move like rigid bodies—domain 1 (114 residues; residues 10–30, 39–102, 234–262) and domain 2 (131 residues; residues 103–233)—after removing flexible regions such as the N-terminal and C-terminal segments as well as the β-sheet (residues 31–38), which were defined at the descendant Nodes 2 and 3 (Figure 2). This information was then used to define the inter-residue distances between the two domains as the input data, excluding the other inter-residue distances that would make the difference between the closed and open forms rather ambiguous.

**Figure 3:**
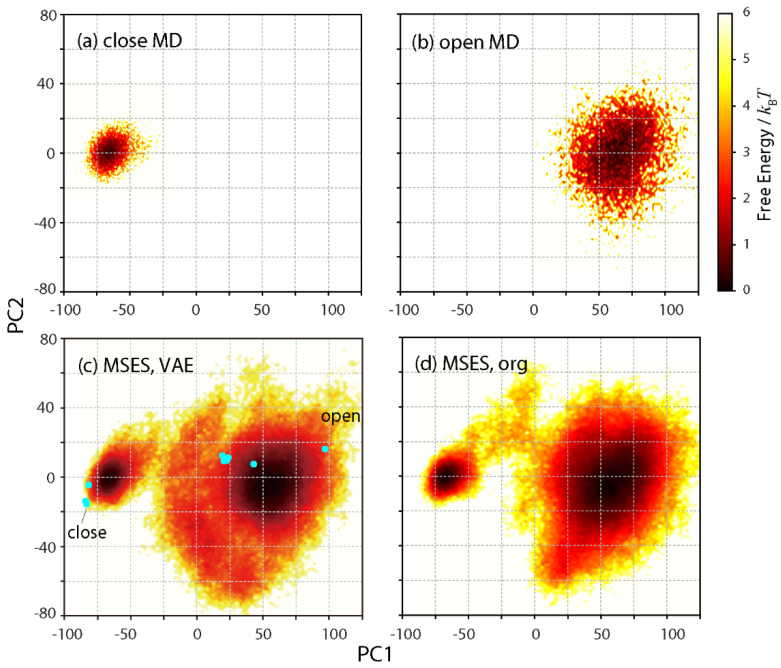
Free energy surfaces in units of *k*_B_*T* along PC1 and PC2 axes that were determined by both the closed and open MD trajectories. (**a**) closed MD, (**b**) open MD, (**c**) VAE-driven MSES, and (**d**) original MSES. In (**c**), all the 11 chains in 7 crystal structures deposited in PDB are also plotted in cyan, including the closed and open structures of the present simulation models.

PC analysis was also performed from the closed and open MD trajectories to determine a set of uncorrelated harmonic distribution functions as linear combinations. The motion of the small domain (domain 1) relative to the large domain (domain 2) was visualized by calculating and diagonalizing the variance–covariance matrix of the interatomic fluctuations in the small domain after superimposing the large domain on a reference coordinate. The first and second PCs, with contributions of 0.93 and 0.04, respectively, were then used to draw the FESs. Figure 3a,b show the distant distributions of the closed and open MDs, mainly along PC1. The difference in the distribution width also indicates much more fluctuation during the open MD than the closed MD.

### 3.2. Generation of Interpolation Data between Closed and Open Forms via Variational Autoendcoder

The VAE network was trained so that the high-dimensional input ***x*** was maximally reconstructed as the output ***y*** through the low-dimensional encoding ***z*** for both the closed and open MD structural ensembles. The number of nodes in the latent space *L* seems to be the most important factor for the success of the dimensional reduction or the choice of proper CVs. To determine the best choice of *L*, the coincidence between ***x*** and ***y*** was then examined as a function of *L*.

**Figure 4:**
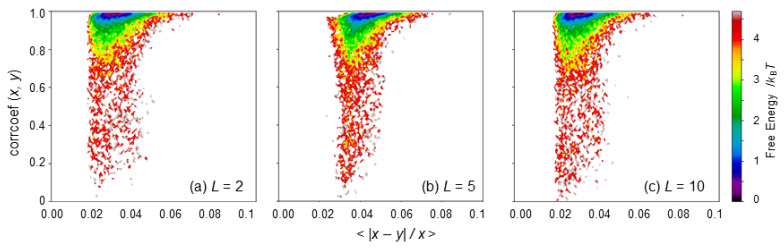
Two-dimensional maps along the averaged relative difference, < |***x*** − ***y*** |/ ***x*** >, and the correlation coefficient between ***x*** and ***y***, where ***x*** and ***y*** are the input and output of VAE (see Figure 1). Free energies in units of *k*_B_*T* are shown. (**a**) *L* = 2, (**b**) *L* = 5, and (**c**) *L* = 10, where *L* is the number of nodes used in the latent layer.

Here, the two quantities—the averaged relative difference, < |***x*** − ***y*** |/ ***x*** >, and the correlation coefficient between ***x*** and ***y***—were used to compare ***x*** and ***y***, where these were calculated for each node of the input ***x*** and the output ***y*** that varied according to the 10,000 closed MD and 10,000 open MD structures, i.e., the number of the calculated data was *N*_inp_ = 14,448. The associated 2D maps were drawn for *L* = 2, 5, and 10 (Figure 4), showing sufficient coincident, i.e., a marginally small difference and a high correlation between ***x*** and ***y*** for the three cases. Note that the data with the correlation coefficient between ***x*** and ***y*** < 0.7 are mostly a consequence of intrinsically very small differences in the averaged inter-residue distances in between the closed and open MDs. A slight improvement or a shift of the peak to the upper left was observed in the 2D map at *L* = 2 relative to the maps at *L* = 5 and 10 (see Figure 4). Furthermore, a smaller *L* is more useful for the visualization and interpretation of the latent space. Thus, *L* = 2 was chosen in this study.

**Figure 5:**
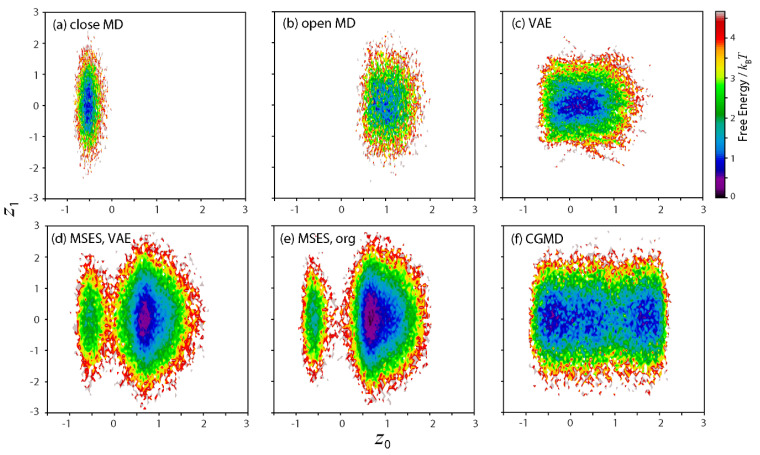
Two-dimensional maps along latent space variables, *z*_0_ and *z*_1_. Free energies in units of *k*_B_*T* are shown. (**a**) closed MD, (**b**) open MD, (**c**) generated time-series data by VAE, (**d**) VAE-driven MSES, (**e**) original MSES, and (**f**) coarse-grained MD, which was applied to the original MSES.

The dynamics seen in the closed and open MD simulations were then examined in the latent space with *L* = 2, i.e., the 2D maps along *z*_0_ and *z*_1_ (Figure 5a,b). The two landscapes show quite distant distributions along *z*_0_, indicating the direction as the CV that most characterizes the difference between the two structural dynamics. In contrast, the distributions along *z*_1_ seem almost the same. This implies that *z*_1_ is unrelated to the structural change between the closed and open forms. In fact, the correlation coefficient of *z*_0_ and *z*_1_ in the training data was very small (0.01).

The data that interpolate the closed and open forms were then generated in the latent space. The resultant dimensional reduction to *L* = 2 made this possible in various ways, compared with the situation when the data interpolation was carried out in the high-dimensional space ***x*** (*N*_inp_ = 14,448). Another advantage of VAE is that the network also allows for the decoding of the generated data to the inter-residue distances in order to be adopted into MSES in a straightforward manner. In order to correspond with the MSES scheme, the generated data must be continuous or a kind of time-series data, ***z*** (*t*). Here, the simplest strategy was taken to fulfill this requirement: (I) a discrete sequence of ***z***, ***z*** (*n*) {*n* = 1, 2,…} was built by randomly taking ***z*** from the pools of both the closed and open MDs one by one, such as ***z*** (1) = ***z***_close_ (1), ***z*** (2) = ***z***_open_ (1), ***z*** (3) = ***z***_close_ (2), ***z*** (4) = ***z***_open_ (2), and so on; and (II) a linear interpolation with equally spaced intervals was performed between ***z*** (*n*) and ***z*** (*n*+1), where the number of intervals was set to 25, corresponding to 25 × 40 ps (i.e., the timestep of the replica exchange in MSES) = 1 ns, the timescale for simulating the open-close structural change. The resultant FES of ***z*** (*t*) covered that of both the closed and open MD simulations (Figure 5c).

### 3.3. VAE-Driven MSES

The time series of the inter-residue distances, ***d***_output_ (t), was calculated by decoding ***z*** (*t*) to the output ***y*** via the VAE network and the calculation of *f*^−1^ (***y***) = ***d***_output_, which was then adopted in the VAE-driven MSES. The Hamiltonian replica exchange using 10 copies resulted in a large average acceptance ratio of the replica exchange (0.32), indicating a high sampling efficiency. The derived FES on PC1 and PC2 covered the whole configuration, including the FESs from both the closed and open MDs as well as the 11 RBP crystal structures (all chains deposited in 7 PDB entries, 1ba2, 1dbp, 1dri, 1drk, 1urp, 2dri, and 2gx6) [41], indicating the success of the present enhanced sampling (Figure 3c). To validate the proposed method, the original MSES adopting the CG model was also performed and compared. The resultant FES on PC1 and PC2 was in reasonable agreement with that of the VAE-driven MSES (Figure 3d). Agreement with other enhanced sampling methods was also seen through umbrella sampling [42] and CGMD [41].

The dynamics in the latent space was also examined. To do this, the structural ensembles from the calculated VAE-driven MSES, the original MSES, and the associated CGMD were encoded to ***z*** via the VAE network. The resultant 2D maps in the latent space are shown in Figure 5d–f. The FES of VAE-driven MSES contained two basins related to the basins from the closed and open MDs (Figure 5d). Since the ligand-unbound form of RBP was simulated, the open basin was intrinsically more stable than the closed basin, which was also seen in the FES along PC1 and PC2 (see Figure 3c). More importantly, this is quite different from the FES from the generated data by simple interpolation between the closed and open MDs, ***z*** (*t*) (see Figure 5c). This result illuminates the usefulness of MD simulations that employ the molecular mechanics force field in refining the generated data in the latent space of VAE. The FES of the original MSES (Figure 5e) was comparable with that of the VAE-driven MSES, although the FES of the CGMD (Figure 5f), which was used to drive the MM conformation in the original MSES, was much broader than the FES from ***z*** (*t*) (see Figure 5c). This result also indicates that the data of the inter-residue distances can be roughly estimated, e.g., based on the minimum requirement for the information on both the closed and open MDs to be included, since the subsequent MSES simulations are used to recover the correct structural ensemble in terms of statistical mechanics.

### 3.4. Prediction of Open Structure via Closed and Holo MDs with VAE-Driven MSES

Finally, an attempt to obtain the RBP structural ensemble in the ligand-unbound form using only the closed structure was made (while keeping the open structure blind), which would lead to the prediction of the open structure. To do this, it was assumed that the dynamics response in the closed form on the ligand unbinding would extrapolate the structural change from the closed to the open forms, which is in accord with the linear response theory stating that the protein structural change is reflected by its intrinsic dynamics [43]. In short, the holo and closed MD trajectories were used to generate the data of the target structural ensemble extending to the open structure, which was accomplished in the latent space of VAE. It must be noted that such data extrapolation can be performed as a rough estimation owing to the subsequent refinement of the structural ensemble by MSES.

**Figure 6:**
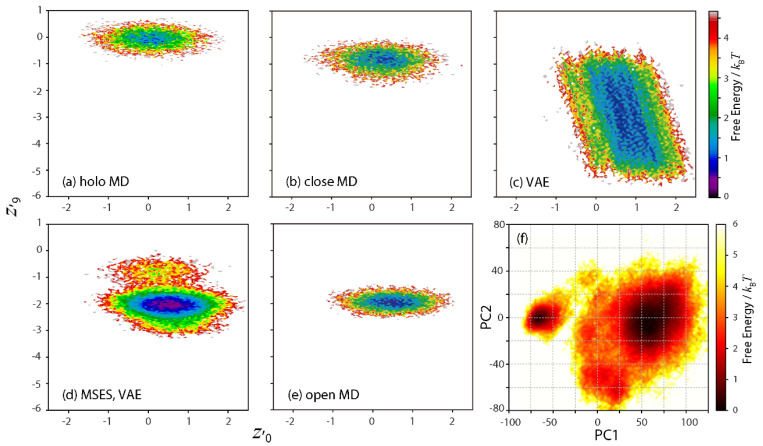
Extrapolation or prediction of the open structure using holo and closed MDs. (**a**–**e**) Two-dimensional maps along two latent space variables, *z*′_0_ and *z*′_9_. Free energies in units of *k*_B_*T* are shown. (**a**) holo MD, (**b**) closed MD, (**c**) generated time-series data by VAE, (**d**) VAE-driven MSES, and (**e**) open MD. In (**f**), the free energy surface calculated from the structural ensemble of VAE-driven MSES along PC1 and PC2 are also shown in units of *k*_B_*T*.

For this purpose, both the holo and the closed MD trajectories were used as the training data to construct the new VAE network. By taking *L* = 10, it was possible to identify two latent space variables (*z*′_0_ and *z*′_9_, where “[*dash*]” indicates the variables in different networks from those previously defined) that characterized the differences between the holo and the closed dynamics (although small differences were seen in the average of the remaining eight *z*′ variables in between the holo and the closed MDs); Figure 6a,b clarifies the shift of the *z*′_0_–*z*′_9_ distribution to the bottom right via ligand unbinding. The need for an increased *L* is probably due to a smaller magnitude and more complication in the difference in dynamics seen in between the holo and the closed MDs, rather than in between the closed and the open MDs.

The time-series data, ***z***′ (*t*), was then generated as follows: (I) the average of ***z***′ was calculated for the holo and closed MDs separately as < ***z***′ >_holo_ and < ***z***′ >_close_, and (II) the ***z***′ distribution extending to the open form, ***z***′_ext_ was built by expanding ***z***′_close_ to the direction of

< ***z***′ >_close_ − < ***z***′ >_holo_, i.e., ***z***′_ext_ = ***z***′_close_ + *α* [< ***z*′** >_close_ − < ***z***′ >_holo_], (4)

where *α* is the scaling factor. In this study, *α* = 5 was determined as the maximum value before the Cα atom distance reached 30 Å between residue 231 and 238, comprising the hinge between the two domains (at which point the hinge is fully extended). ***z***′ (*t*) was then generated by connecting the distributions of ***z***′_close_ and ***z***′_ext_, in the same way as the previous interpolation between ***z***_close_ and ***z***_open_ (see Section 3.2). The 2D map of the derived ***z***′ (*t*) certainly expanded toward the bottom right from the distribution of the closed MD (Figure 6c).

The VAE-driven MSES simulation was then carried out by adopting ***z***′ (*t*). The 2D map in the latent space calculated from the derived structural ensemble (Figure 6d) contained two basins related not only to the closed MD but also to the open MD (Figure 6e), demonstrating the structural sampling in the open form without using any information in the open structure. Note that the distribution of ***z***′ (*t*) (see Figure 6c) was much broader than that of the MSES structural ensemble (see Figure 6d). This actually led to a smaller average acceptance ratio of the Hamiltonian replica exchange (0.26), using the same number of copies and *k*_MMCG_ values, and indicating a slightly decreased sampling efficiency. Nevertheless, the derived FES on PC1 and PC2 (Figure 6f) was almost the same as that from the VAE-driven MSES using the data of the closed and open MDs (see Figure 3c). It can then be concluded that the ability of the VAE-driven MSES to refine the extrapolated data in the latent space as a rough estimation, i.e., using a structural criterion that the inter-domain hinge does not distort, allowed for the prediction of the RBP structural dynamics in the ligand-unbound form, or of the open structure as the most stable state, using only the MD simulations in the closed form.

## 4. Conclusions

In this study, an extension of the MSES simulation using VAE, a machine learning approach, was proposed and applied to the domain motion of RBP. This method allowed the dynamics in the reduced subspace generated by VAE to perform an enhanced sampling of an all-atom protein structure. Here, the closed and open MD trajectories of RBP were used as the input of the training data after normalizing the residual-residue distances as the structural features. The Motion Tree constructed from the two trajectories was utilized to define the dynamic domains and the inter-domain residue pairs. The trained VAE model could characterize the difference in the structural dynamics of the closed and open forms in the encoded latent space. The interpolated data were then generated in the low-dimensional latent space by simply connecting the distributions of the closed and open MDs, which were then decoded to the time-series data of the inter-residue distances and used to drive the structural sampling at an atomistic resolution via the MSES scheme. The derived structural ensemble was found to be in reasonable agreement with that from the original MSES adopting the CG model, thus validating the proposed VAE-driven MSES.

The dynamics in the latent space was then examined, since the sampling efficiency of the MSES simulation would rely on the dimensional reduction or the proper choices of CVs via VAE. The distribution calculated from the MSES structural ensemble after encoding this in the latent space contained two basins related to the closed and open forms, reflecting the 2D FES on PC1 and PC2. In contrast, the generated data in the latent space yielded a single basin combining the two closed and open basins. The difference in the latent space distributions indicates that the subsequent MSES can accurately refine the consequent structural ensemble on the basis of statistical mechanics together with the molecular mechanics force field. In this sense, allowing for a rough estimation of the generated data via VAE is the advantage of the present method, although it is rather time-consuming to perform additional MD simulations after the VAE network optimization. This is because it is still difficult for solely a deep neural network to complete the prediction of the whole structural dynamics, especially for comparative systems such as the binding of different ligands and in various kinds of surrounding solvents. Essentially, the prediction of the unknow open structure was demonstrated to be possible by using only the closed MDs with and without the bound ligand and by extrapolating the data related to the open-close motion.

## Data Availability

Data supporting the reported results and the source code on the VAE implementation are available from the author upon request.
